# Tau mediates the reshaping of the transcriptional landscape toward intermediate Alzheimer’s disease stages

**DOI:** 10.3389/fcell.2024.1459573

**Published:** 2025-01-03

**Authors:** Giacomo Siano, Martina Varisco, Marco Terrigno, Congwei Wang, Arianna Scarlatti, Vincenzo Iannone, Marco Groth, Marie-Christine Galas, Jeroen J. M. Hoozemans, Alessandro Cellerino, Antonino Cattaneo, Cristina Di Primio

**Affiliations:** ^1^ Laboratory of Biology, BIO@SNS, Scuola Normale Superiore, Pisa, Italy; ^2^ Istituto di Neuroscienze, Consiglio Nazionale delle Ricerche, Pisa, Italy; ^3^ Roche Pharma Research and Early Development, Roche Innovation Center Basel, Basel, Switzerland; ^4^ CF Next-Generation Sequencing, Leibniz Institute on Ageing – Fritz Lipmann institute, Jena, Germany; ^5^ University of Lille, Institut national de la santé et de la recherche médicale, CHU-Lille, Centre national de la recherche scientifique, LilNCog-Lille Neuroscience & Cognition, Lille, France; ^6^ Department of Pathology, Amsterdam Neuroscience, Amsterdam University Medical Centers, Amsterdam, Netherlands; ^7^ Leibniz Institute on Ageing, Fritz Lipmann institute, Jena, Germany

**Keywords:** tau, Alzheimer’s disease, gene expression, chromatin remodeling, epigenetic markers, AD cellular model

## Abstract

**Introduction:**

Recent research revealed that Tau plays critical roles in various neuronal functions. We previously demonstrated that destabilization and nuclear delocalization of Tau alter the expression of glutamatergic genes, mediating early neuronal damage.

**Methods:**

In this study, we discovered that changes in Tau availability are linked to global alterations in gene expression that affect multiple neuronal pathways. Comparison with the human temporal region showed that the Tau-dependent modulation of gene expression closely resembles the intermediate stages of Alzheimer’s disease (AD) that precede the definitive pathological condition.

**Results:**

Furthermore, we identified the chromatin remodeling pathway as being significantly affected by Tau in both our cellular model and AD brains, with reductions in heterochromatin markers. Our findings indicate that Tau is able to globally affect the neuronal transcriptome and that its subcellular unbalance changes gene expression in the intermediate stages of AD development. In addition, we found that the chromatin architecture is affected by Tau during the progression of AD.

**Discussion:**

These results provide new insights into the molecular mechanisms underlying early stages of AD development and highlight the central role of Tau and the contribution of nuclear Tau in this process.

## 1 Introduction

The microtubule-associated protein Tau is a key factor in the maintenance of neuronal homeostasis and cytoskeletal stability (Guo et al., 2017). During the early stages of tauopathies, Tau exhibits specific characteristics: its expression levels increase, its soluble fraction expands, and it progressively forms oligomers and aggregates that result in neuronal damage and cell death (Kurbatskaya et al., 2016; Wang and Mandelkow, 2016; Han et al., 2017). While aggregates have traditionally been considered the primary cause of neurodegeneration; recent discoveries regarding Tau nuclear functions suggest a broader and more intricate role in neuronal homeostasis changes (Sotiropoulos et al., 2017; Siano et al., 2021). Previous observations hint that Tau has a connection with transcription regulation (Mansuroglu et al., 2016; Rousseaux et al., 2016; Benhelli-Mokrani et al., 2018; Guo et al., 2018; Sun et al., 2018; Rico et al., 2021). Recent studies reported that the progression of AD in the prefrontal cortex is the result of a multi-scale disruption of genome organization. This evidence indicates that the chromatin landscapes are dynamically altered, causing the deregulation of transcriptomic pathways, resulting in cell-type specific dysfunction. (Dileep et al., 2023; Gazestani et al., 2023; Xiong et al., 2023). A connection between Tau and chromatin structure has been previously described since Tau directly interacts with TRIM28, a key protein involved in histone modifications. Indeed, we recently demonstrated that Tau causes the delocalization of the chromatin remodeler HDAC1, an event associated with the reduction of heterochromatin (Lagger et al., 2002; Siano et al., 2023), suggesting a possible mechanism mediating the Tau-dependent epigenetic and transcriptional alteration. Notably, Tau interacts with chromatin, playing a crucial role in genomic stability by safeguarding DNA from damage and maintaining nucleolar organization under stress conditions (Sjöberg et al., 2006; Rossi et al., 2008; Violet et al., 2014; Mansuroglu et al., 2016; Maina et al., 2018; Colnaghi et al., 2020). Additionally, Tau is implicated in transposon activation, a process closely linked to Alzheimer’s disease (AD) (Guo et al., 2018; Sun et al., 2018). Our recent study revealed that nuclear Tau alters the expression of glutamatergic genes, resulting in toxic hyperexcitability during the early stages of AD. Intriguingly, aggregation of Tau prevents this mechanism, reducing glutamatergic deregulation (Siano et al., 2020b).

In this study, we demonstrate that Tau’s influence on the neuronal transcriptome includes reshaping the chromatin landscape during the early and intermediate stages of AD. Specifically, we show that Tau leads to widespread epigenetic alterations, including a progressive Tau-dependent loss of heterochromatin markers, both in a neuroblastoma SH-SY5Y cell line model and in human AD brain tissues. These findings indicate that Tau plays a pivotal role in the early transcriptional dysregulation seen in AD and reveal a previously underappreciated mechanism whereby Tau contributes to disease pathology through its impact on chromatin remodeling. This novel insight into Tau’s nuclear function adds a critical dimension to our understanding of AD progression, suggesting that Tau’s role in reshaping the transcriptional landscape may be a key driver of early AD pathology.

By employing a high-resolution transcriptomic approach, our findings highlight that the transcriptional changes mediated by Tau closely align with intermediate AD stages, suggesting that Tau-driven chromatin remodeling could be a major factor in the onset and exacerbation of AD. This study thus reveals a new and crucial function for Tau in the progression of AD, offering potential new avenues for therapeutic intervention.

## 2 Materials and methods

### 2.1 Cell culture, transfection and differentiation

SH-SY5Y neuroblastoma cells were maintained in DMEM/F12 (Gibco) supplemented with foetal bovine serum (FBS) and penicillin/streptomycin. For the transcriptomic experiments, 2 × 10^5^ cells were plated in p30 wells and transfected the day after seeding with Lipofectamine 2,000 according to the manufacturer’s instructions. The day after transfection, cells were differentiated by 10 µM retinoic acid (Sigma‒Aldrich) for 5 days, followed by 50 ng/mL BDNF (Alomone Labs) for 15 days in serum-free DMEM/F12 medium. After differentiation, cells were collected for Western blotting and RNA extraction. For Tau overexpression, the cDNA encoding Tau isoform D (383 aa) was cloned into the BglII site of pcDNA3.1, and an empty pcDNA3.1 vector was used as a control as previously described (Siano et al., 2019b).

### 2.2 Post-mortem brain samples

Fresh-frozen human post-mortem brain samples were collected at the pathology departments of VUmc (Amsterdam, Netherlands), with approval granted by VUmc for their use in this study. All samples were treated with informed consent and ethical considerations; that is, tissue was collected post-mortem from donors from whom full consent had been obtained during life to conduct a brain autopsy for research purposes and all methods were carried out in accordance with relevant guidelines and regulations. Tissues from both male and female donors were used in this study. There was no influence or association of sex on the findings reported.

### 2.3 Western blot analysis

For Western blot (WB) analysis, experiments were performed as previously described (Siano et al., 2020a). In brief, total protein extracts were prepared in lysis buffer supplemented with protease and phosphatase inhibitors (Roche). Proteins were quantified by bicinchoninic acid (BCA) assay (Thermo Fisher Scientific). Twenty micrograms of total proteins were loaded for each sample. The proteins were separated by SDS–PAGE and electroblotted onto Hybond-C-Extra (Amersham Biosciences) nitrocellulose membranes. The membranes were blocked with 5% skimmed milk powder in TBS and 0.1% Tween 20. The antibodies for WB were as follows: mouse anti-Tau (Tau13) 1:1,000 (Santa Cruz Biotechnology), rabbit anti-HP1α 1:500 (Abcam), rabbit anti-H3K9me3 1:500 (Abcam), rabbit anti-eIF1α 1:1,000 (Cell Signaling), rabbit anti phospho-eIF1α 1:1,000 (Cell Signaling), mouse anti-GAPDH 1:10,000 (Santa Cruz Biotechnology), HRP-conjugated anti-mouse 1:1,000 (Santa Cruz Biotechnology), and HRP-conjugated anti-rabbit 1:1,000 (Santa Cruz Biotechnology). Western blot quantification was performed using ImageJ software.

### 2.4 Immunofluorescence

For Immunofluorescence (IF) experiments *in vitro*, cells were fixed with ice-cold 100% methanol for 5 min. The cells were permeabilized (PBS, 0.1% Triton X-100), blocked (1% wt/vol BSA) and incubated with primary and secondary antibodies. The slides were mounted with VECTASHIELD mounting medium (Vector Laboratories). The antibodies for IF were as follows: mouse anti-Tau (Tau13) 1:500 (Santa Cruz Biotechnology), rabbit anti-HP1α 1:250 (Abcam), rabbit anti-H3K9me3 1:500 (Abcam), mouse anti-Tau AT8 1:250 (Invitrogen), anti-NeuN (Boster Biological Technology) 1: 300, anti-mouse Alexa Fluor 633, and anti-rabbit Alexa Fluor 488 (Life Technologies). Nuclear staining was performed by incubation for 15 min with DAPI. Images were acquired on a Zeiss Laser Scanning (LSM) 880 confocal microscope (Carl Zeiss, Jena, Germany) supplied with GaAsP (gallium:arsenide:phosphide) detectors. The samples were viewed with a 63X Apochromat oil immersion (1.4 NA) DIC objective. Whole-cell images were acquired with a z-stack series with 0.5 µm intervals and summed with the z-projection tool from Fiji. Chromatin marker fluorescence was analysed by Fiji software.

Freshly frozen brain tissue was sectioned at 10 μm using a cryostat (Thermo Fisher Scientific). The brain slices were fixed in ice-cold acetone for 5 min before being rinsed twice with PBS and twice with PBS-T and blocked in animal-free blocker for 30 min. The tissue sections were treated for 1 h at room temperature with primary antibodies diluted in blocking solution, rinsed three times with PBS-T, and then incubated with secondary antibodies for 1 h at room temperature. Following a PBS-T wash, the slices were incubated with PureBlu DAPI (Bio-Rad, Cat# 1351303) for 3 min before being mounted with ProLong Gold antifade mounting media (Thermo Fisher Scientific, Cat# P36934). Images were scanned using an Olympus SLIDEVIEW VS200 slide scanner.

ImageJ software was used to analyse immunofluorescence images. Image5D tool followed by Z Projection was employed for z-stack images. For HP1α and H3k9me3 fluorescence quantification, ROI of cellular nuclei has been drawn by ImageJ and mean fluorescence quantified in Tau positive cells or in Ctrl cells and corrected for the total fluorescence intensity. In AD brains, H3K9me3 fluorescence was measured in NeuN and AT8 positive cells after background subtraction and corrected for the total fluorescence intensity. In control BS1 and BS2 samples, AT8 signal was undetectable or very low. In AD samples from BS3 to BS6 we detected a significant and increasing AT8 signal. H3K9me3 fluorescence in NeuN/AT8-positive cells was compared to NeuN-positive cells in BS1/2 samples. An average of 55 cells were analysed for each brain sample. For imaging analysis, images have not been manipulated. For representative images in the text, Brightness and Contrast plugin was used homogeneously for the different experimental groups to clarify measured differences.

### 2.5 Statistical analysis

For WB and IF analyses, the non-parametric Kruskal–Wallis test was used. All results are shown as the mean ± standard error of the mean (SEM). For WB experiments, N ≥ 3 independent biological experiments were performed. For IF experiments on neuroblastoma cells: N ≥ 30; each biological replicate corresponded to one cell. For human brain sample analyses, N ≥ 4. Significance is indicated as * for *p* < 0.05, ** for *p* < 0.01, *** for *p* < 0.001 and **** for *p* < 0.0001.

### 2.6 RNA extraction and RNA-seq

Two experimental groups were compared: the control and Tau^WT^ overexpression groups. RNA was extracted with a NucleoSpin (Macherey-Nagel) RNA extraction kit according to the manufacturer’s instructions. Sequencing of RNA samples was performed using Illumina’s next-generation sequencing methodology (Bentley et al., 2008). In detail, total RNA was quantified and quality checked using Bioanalyzer 2,100 instrument in combination with RNA 6000 nano kit (both Agilent Technologies). Libraries were prepared from 800 ng of input material (total RNA) using NEBNext Ultra II Directional RNA Library Preparation Kit in combination with NEBNext Poly(A) mRNA Magnetic Isolation Module and NEBNext Multiplex Oligos for Illumina (Index Primers Set 1/2/3/4) following the manufacturer’s instructions (New England Biolabs). Quantification and quality check of libraries was done using an Bioanalyzer 2,100 instrument and DNA 7500 kit (Agilent Technologies). Libraries were pooled and sequenced in two lanes on a HiSeq 2,500. System runs in 51 cycle/single-end/high-throughput (SBS reagent v3) mode. Sequence information was converted to FASTQ format using bcl2fastq v1.8.4.

### 2.7 RNA sequencing data analysis

The reads were mapped onto the genome (Ensembl: GRCh38.92 (Zerbino et al., 2018)) with Tophat v2.1 (Langmead et al., 2009; Kim et al., 2013) using parameter--no-convert-bam--no-coverage-search -x 1 -g 1: on average, 80% of all reads were mapped univocally. The reads per gene were counted using featureCounts v1.5.0 (Liao et al., 2019). Read counts were introduced in the statistical environment R (v.3.4.1) for further processing. Read counts were normalized for reads per sample (RPM) and transcript length (RPKM) (Mortazavi et al., 2008). DEGs were defined by an FDR cut-off of <0.1 in the statistical tests of all 3 R packages, edgeR (Robinson et al., 2010), DESeq (Anders and Huber, 2010) and baySeq (Hardcastle and Kelly, 2010), and by an absolute log2FC > 0.5. The 300 most variant genes were selected for the PCA plot with the prcomp function in the R ‘stats’ package (R v4.1.3) after variance stabilization in DESeq2 v1.34.0 (Anders and Huber, 2010). The EnhancedVolcano package v1.12.0 was used for the volcano plot (Blighe et al., 2018). The dataset is available upon request (dataset reference number GSE239956).

GO analysis of the DEGs was performed by ORA with the GO Biological Process annotations from the GO. db package v3.14.0 (Carlson, 2019). ORA was performed by a right-sided hypergeometric test, and terms with BH-adjusted *p* values <0.05 were considered significant. WebGestalt was used to summarize GO entries with the affinity propagation method (Wang and Reddy, 2017). For KEGG pathway enrichment, pathways were retrieved using the KEGGREST package v1.34.0 (Tenebaum and Maintainer, 2023), and the Wilcoxon test was performed for each pathway on the merged set of up- and downregulated DEGs. Pathways with a P value <0.05 and number of genes >10 are reported in the table.

GSEA was performed on all the genes ranked by log2FC using the fgsea R package v1.31.0 (Korotkevich et al., 2023) on the msigdbr GO Biological Process database v7.5.1 (Dolgalev, 2022), with parameters minSize = 15 and maxSize = 500. The top 10 upregulated and top 10 downregulated pathways were displayed with the plotGseaTable function in fgsea (Korotkevich et al., 2023).

### 2.8 Gene expression comparison with the human dataset

For transcriptome meta-analysis, public transcriptomic datasets were sourced from the GEO platform hosted on NCBI (https://www.ncbi.nlm.nih.gov/geo/). The GSE84422 dataset (Wang et al., 2016), which includes data from 1,053 *postmortem* brain samples across 19 brain regions from a total of 125 patients dying at different AD stages, with 50–60 subjects per brain region, was used. The patients were clustered by AD severity into four groups, the normal (n = 110), posAD (n = 110), proAD (n = 83) and defAD (n = 163) groups, according to their clinical dementia rating (CDR), Braak NFT score, sum of NFT density across different brain regions, average neuritic plaque density and average Consortium to Establish a Registry for Alzheimer’s Disease (CERAD) score. The R packages GEOquery (Davis and Meltzer, 2007), Rcpp v1.0.12 (Eddelbuettel and François, 2011) and tidyselect v1.2.1 were used to import datasets and create subsets by diagnosis and sample type or brain region. To compare different platforms, platform-specific IDs were converted into Entrez Gene IDs using the corresponding R annotation databases from Bioconductor, available via bioMart v3.8 (Durinck et al., 2009). Multiple platform IDs matching the same gene were summarized in a single entry with gene expression as the mean of probe expression. Probes mapping on the same gene with a discordant logFC were excluded from the analysis to preserve more reliable data. Differential expression analysis of microarray data was performed with the R package limma v3.8 (Ritchie et al., 2015). An FDR-adjusted *p* value <0.1 was set as the cut-off for differential expression.

GO terms associated with genes were retrieved using the GO Biological Process annotations from the GO. db package v3.14.0 (Carlson, 2019). The direction and magnitude of the logFC of genes in selected transcription- and chromatin-related GO pathways were compared between SH-SY5Y and patients in dataset GSE84422 by Pearson correlation. All DEGs in the SH-SY5Y transcriptome were included in the analysis.

To assess the closest AD stage to the SH-SY5Y cell model, the logFC of each of the SH-SY5Y DEGs was compared to data of patients from GSE84422 at different AD stages and visualized with the ComplexHeatmap R package v 2.10.0 (Gu et al., 2016). The global logFC correlation of SH-SY5Y DEGs with AD stages was displayed by sample-wise correlation heatmap with ComplexHeatmap (Gu et al., 2016).

### 2.9 GO on common genes between tau-overexpressing SH-SY5y and hippocampus proAD transcriptome

GO was performed on genes differentially expressed in our transcriptomic data in Tau-overexpressing SH-SH5y (absolute log FC > 0.5 and FDR-adjusted *p* value <0.1) and with a log FC in the same direction in the hippocampus of proAD brains from GSE84422 (absolute log FC > 0.1). Upregulated and downregulated genes were analyzed separately for overrepresentation of Biological Process terms from the R package GO. db v3.14.0, using the hypergeometric test implemented in the hyperGTest function from the GOstats R package v2.60.0. Pathways with *p*-value <0.05 were deemed statistically significant. The enrichment and log10-transformed *p*-value of the significantly overrepresented pathways related to nuclear terms (transcription, chromatin, chromosome, histone, DNA) were displayed with ggplot2.

### 2.10 Overlap DEGs from the transcriptome of tau-overexpressing SH-SY5y with changes in chromatin marks in AD brains

Up-, downregulated and non-significant genes from transcriptomic data in Tau-overexpressing SH-SY5y cells were compared to the genes with changes in histone acetylation on H3K27, H3K9 and H3K122 in AD brains from published datasets: 1. Marzi et al., GEO accession number GSE102538 (H3K27ac) (Marzi et al., 2018); 2. Patel et al., accession numbers in supplementary tables (H3K27ac) (Patel et al., 2023);, 3. Nativio et al., GEO accession number GSE153875, GSE130746 (H3K27ac, H3K9ac, H3K122ac) (Nativio et al., 2020); 4. Klein et al., accession numbers in supplementary tables (H3K9ac) (Klein et al., 2019).

Significant overlaps were calculated by hypergeometric test for overrepresentation with the phyper R base function, with BH-adjusted *p*-value <0.05 as the significance threshold. The log10-transformed adjusted *p*-value of significant overlaps and their enrichment in the up- or downregulated set from our transcriptome with respect to the genome-wide baseline were displayed with ggplot2.

The number of downregulated and upregulated differential genes in our dataset that overlap with changes in acetylation in any dataset was displayed in an upset plot using the ComplexUpset R package v1.3.3. The ‘down’ category includes genes with significantly reduced acetylation in all the datasets reporting a change in gene acetylation, and the ‘up’ category includes genes with consistently increased acetylation. The ‘any’ set includes genes with discordant acetylation changes across datasets.

## 3 Results

### 3.1 Tau alters global gene expression in neuronal cellular model

During the progression of Alzheimer’s disease, the amounts of total and soluble Tau protein progressively increase even before significant hyperphosphorylation or aggregation, suggesting an early imbalance of Tau cellular levels (Kurbatskaya et al., 2016; Han et al., 2017). We previously observed that the increase in soluble Tau leads to its accumulation in the nucleus, which alters the expression of disease-related genes in the early stages (Siano et al., 2019a; Siano et al., 2019b; Siano et al., 2020b). Based on this evidence, we investigated whether Tau could directly modulate nuclear pathways, with a particular focus on the early stages.

**FIGURE 1:**
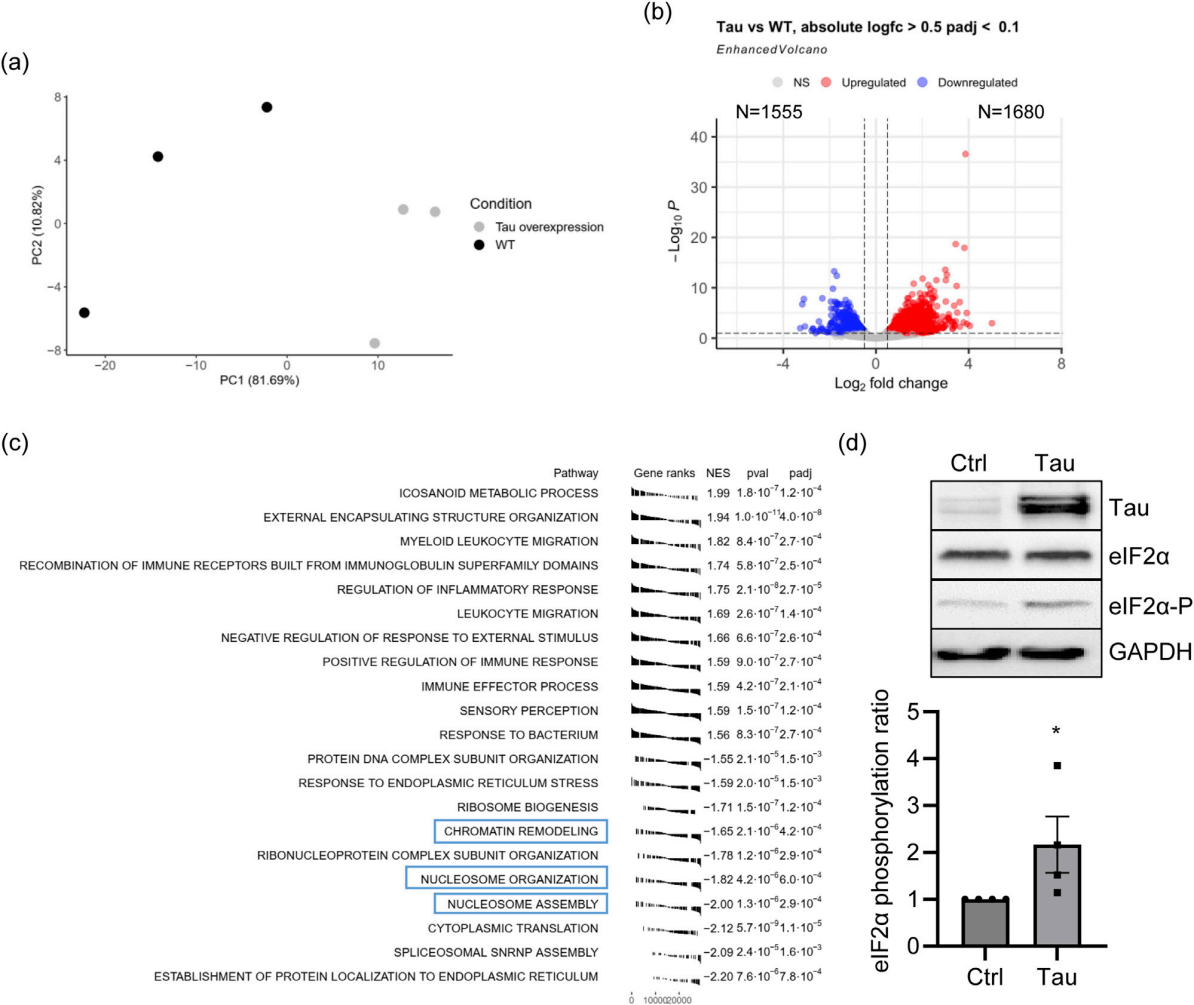
Tau overexpression in neuroblastoma cell lines leads to transcriptomic alterations. **(A)** PCA of proAD-mimic and control cells. **(B)** Volcano plot of DEGs. Red indicates upregulated genes, and blue indicates downregulated genes with |logFC| > 0.5 and FDR <0.1. Supplementary Table S1 for additional data **(C)** GSEA analysis (Biological Process) of the proAD-mimic transcriptome. **(D)** WB and relative quantification of differentiated SH-SY5Y cells overexpressing Tau (proAD-mimic; Tau) and control (Ctrl) cells. Tau, eIF2α and phosphorylated eIF2α (eIF2α-P) were measured with respect of the housekeeping GAPDH. The ratio between eIF2α-P and eIF2α has been reported in the graph. N = 4; *p < 0.05.

We overexpressed Tau 0N4R, one of the most abundant Tau isoforms in the adult human brain, in differentiated neuroblastoma cells, a cellular model commonly used to study molecular mechanisms in tauopathies (Bell and Zempel, 2022; Siano et al., 2023). The global transcript expression was quantified by RNA sequencing (RNA-seq) (dataset reference number GSE239956). Principal component analysis (PCA) confirmed that the increased expression of Tau is the main factor contributing to variation in the global transcriptome profile (Figure 1A). RNA-seq data analysis revealed 3,235 differentially expressed genes (DEGs) between Tau-overexpressing and control cells (Figure 1B). Upon Tau overexpression, 1,680 DEGs were upregulated and 1,555 were downregulated (Figure 1B). KEGG analysis identified several pathways significantly overrepresented among the DEGs, and the most significant are listed in Table 1. Several terms were related to neuronal and synaptic pathways, confirming our previous observation that Tau modulates the expression of neuronal genes and might participate in triggering synaptic dysfunction occurring in early AD stages (Siano et al., 2019b). GSEA analysis showed pathways mainly related to immune response, signaling and protein homeostasis (Figure 1C), a result further supported by the GO analysis of upregulated and downregulated DEGs (Supplementary Figure S1, S2). Several overrepresented GSEA pathways were associated with nucleosome organization and chromatin remodeling, suggesting that altered levels of Tau protein could also affect these nuclear mechanisms. Of note, the overexpressed Tau is as soluble as the endogenous one, supporting that Tau levels, rather than its aggregation, are responsible for the observed effects (Supplementary Figure S3). In the transcriptome analysis, the protein homeostasis pathways significantly arise. We measured the levels of phospho-eIF2α after Tau overexpression as a well-known marker of response to ER stress and translational mechanisms (Clemens, 2001; Radford et al., 2015; Gerakis and Hetz, 2018; Shacham et al., 2021). We assessed the ratio between eIF2α-P and total eIF2α in our cellular model and we observed a significant increase after Tau overexpression confirming the reliability of the analysis and of the model (Figure 1D).

**TABLE 1:** Gene pathways altered by Tau. KEGG pathways of genes altered by Tau in neuroblastoma cell lines. Supplementary Table S2 for additional data.

Id	KEGG pathway	N	Pvalue
hsa04974	Protein digestion and absorption	20	1.38E-05
hsa04141	Protein processing in endoplasmic reticulum	53	2.04E-04
hsa04261	Adrenergic signaling in cardiomyocytes	33	2.61E-04
hsa04024	cAMP signaling pathway	45	2.82E-04
hsa04512	ECM-receptor interaction	22	2.86E-04
hsa04918	Thyroid hormone synthesis	18	8.21E-04
hsa04925	Aldosterone synthesis and secretion	22	9.15E-04
hsa04022	cGMP-PKG signaling pathway	28	1.04E-03
hsa05414	Dilated cardiomyopathy	25	1.25E-03
hsa04919	Thyroid hormone signaling pathway	19	1.61E-03
hsa04933	AGE-RAGE signaling pathway in diabetic complications	25	2.04E-03
hsa05031	Amphetamine addiction	18	2.08E-03
hsa04926	Relaxin signaling pathway	26	2.55E-03
hsa05206	MicroRNAs in cancer	40	2.71E-03
hsa05412	Arrhythmogenic right ventricular cardiomyopathy	22	4.72E-03
hsa05214	Glioma	14	5.78E-03
hsa04020	Calcium signaling pathway	49	7.90E-03
hsa04928	Parathyroid hormone synthesis, secretion and action	27	8.13E-03
hsa04151	PI3K-Akt signaling pathway	66	9.06E-03
hsa04972	Pancreatic secretion	21	9.53E-03
hsa05165	Human papillomavirus infection	64	9.58E-03
hsa05210	Colorectal cancer	19	9.79E-03
hsa04510	Focal adhesion	49	1.24E-02
hsa04730	Long-term depression	14	1.61E-02
hsa04961	Endocrine and other factor-regulated calcium reabsorption	14	1.66E-02
hsa04814	Motor proteins	36	1.69E-02
hsa04727	GABAergic synapse	18	1.76E-02
hsa04970	Salivary secretion	16	1.82E-02
hsa04068	FoxO signaling pathway	27	1.85E-02
hsa00564	Glycerophospholipid metabolism	20	2.01E-02
hsa04360	Axon guidance	44	2.07E-02
hsa05410	Hypertrophic cardiomyopathy	25	2.44E-02
hsa04921	Oxytocin signaling pathway	31	2.44E-02
hsa01521	EGFR tyrosine kinase inhibitor resistance	17	2.87E-02
hsa04915	Estrogen signaling pathway	24	3.09E-02
hsa04613	Neutrophil extracellular trap formation	16	3.14E-02
hsa04218	Cellular senescence	31	3.31E-02
hsa04927	Cortisol synthesis and secretion	15	3.61E-02
hsa05223	Non-small cell lung cancer	15	3.69E-02
hsa05166	Human T-cell leukemia virus 1 infection	43	3.76E-02
hsa05146	Amoebiasis	16	4.18E-02
hsa04350	TGF-beta signaling pathway	29	4.35E-02
hsa04611	Platelet activation	20	4.39E-02
hsa04270	Vascular smooth muscle contraction	27	4.43E-02
hsa05200	Pathways in cancer	96	4.53E-02
hsa00970	Aminoacyl-tRNA biosynthesis	11	4.56E-02
hsa04216	Ferroptosis	12	4.59E-02
hsa05034	Alcoholism	27	4.65E-02
hsa04060	Cytokine-cytokine receptor interaction	28	4.79E-02

### 3.2 Tau-dependent alteration of global gene expression *in vitro* resembles proAD conditions

**FIGURE 2:**
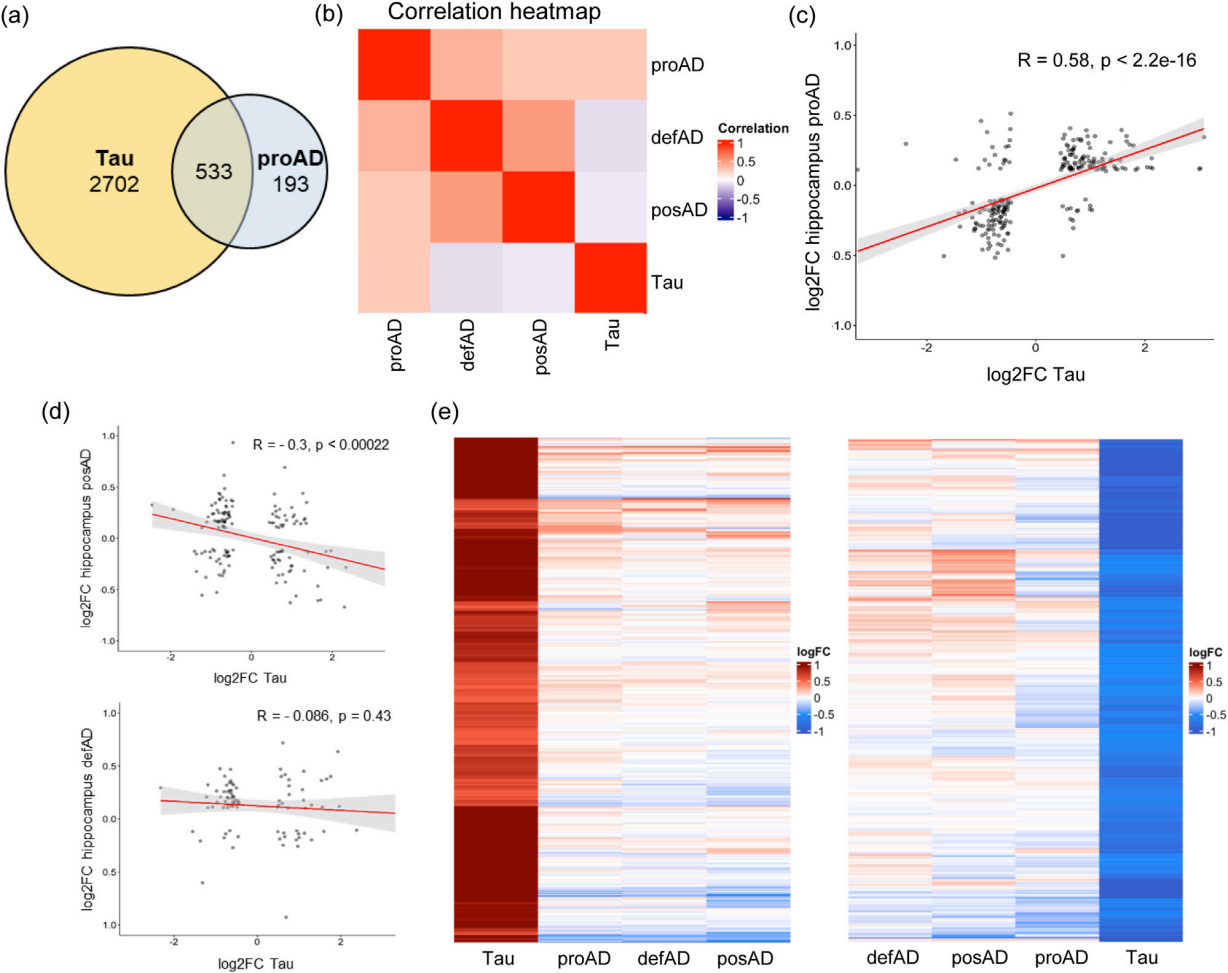
Tau-dependent transcriptome alteration correlates with the proAD stage in the human temporal region. **(A)** Common altered genes between proAD-mimic cells and proAD human brains. **(B)** Correlation heatmap of proAD-mimic model and AD brains stages. **(C)** Scatter plot representing the log2FCs of genes in proAD-mimic cells (*x*-axis) and proAD human brains (*y*-axis). **(D)** Scatter plot representing the log2FCs of genes in proAD-mimic cells (*x*-axis) and posAD or proAD human brains (*y*-axis). **(E)** Heatmap of upregulated and downregulated genes in proAD-mimic cells and posAD, proAD and defAD brains. Biological groups are ordered depending on their correlation profile proximity (proAD-mimic and proAD brain are always the closest profiles).

To investigate whether Tau-dependent transcriptomic alteration replicates the dysregulation of genes in particular AD phases, we compared our RNA-seq data with the human brain transcriptome in various AD stages from a published dataset, GSE84422 (GPL97 platform). In this dataset AD brain samples are structured into 3 groups based on the integration of several diagnostic parameters (see Mat and Met): possible AD (posAD), probable AD (proAD) and definitive AD (defAD), representing early, intermediate, and late phases, respectively (Wang et al., 2016). The proAD condition significantly overlapped our dataset (Figure 2A). A correlation analysis with different AD stages in the temporal region revealed a positive correlation specifically between the cellular model and the proAD, while low or negative correlation with posAD and defAD respectively (Figure 2B). Hereinafter, the *in vitro* model of SH-SY5Y cells overexpressing Tau is referred to as proAD-mimic. By plotting the log fold changes (log2FCs) of proAD-mimic DEGs and posAD, proAD, and defAD, we found a positive correlation between our dataset and proAD, as expected, with approximately 75% of common genes displaying the same direction of regulation (R = 0.58 and *p* < 2.2e-16; Figure 2C). According to this analysis, the Tau-dependent gene alterations in the proAD-mimic represented a developing intermediate AD condition in the hippocampus. In addition, there was a negative correlation for posAD and no correlation for defAD (posAD: R = −0.3 and *p* < 0.00022; defAD: R = −0.086 and *p* value = 0.43; Figure 2D), with approximately 60%–65% common genes in the second and fourth quadrants. The heatmap representation of DEGs showed a higher sensitivity of the cellular model probably due to the differences in the techniques and in the cellular composition of the culture with respect to the multicellular composition of the brain. The heatmap restricted to upregulated and downregulated DEGS of the proAD-mimic, compared to posAD, proAD and defAD, indicates that the proAD dataset is the closest to the proAD-mimic, showing a comparable gene expression profile (Figure 2E). In addition, posAD and defAD present a comparable profile (Figure 2E), suggesting a bell-shaped trend of transcriptomic alteration during AD progression, as previously reported for synaptic pathways (Siano et al., 2020b; Amato et al., 2024).

### 3.3 HP1α and H3K9me3 reduction occurs early in both cellular model and human brains

**FIGURE 3:**
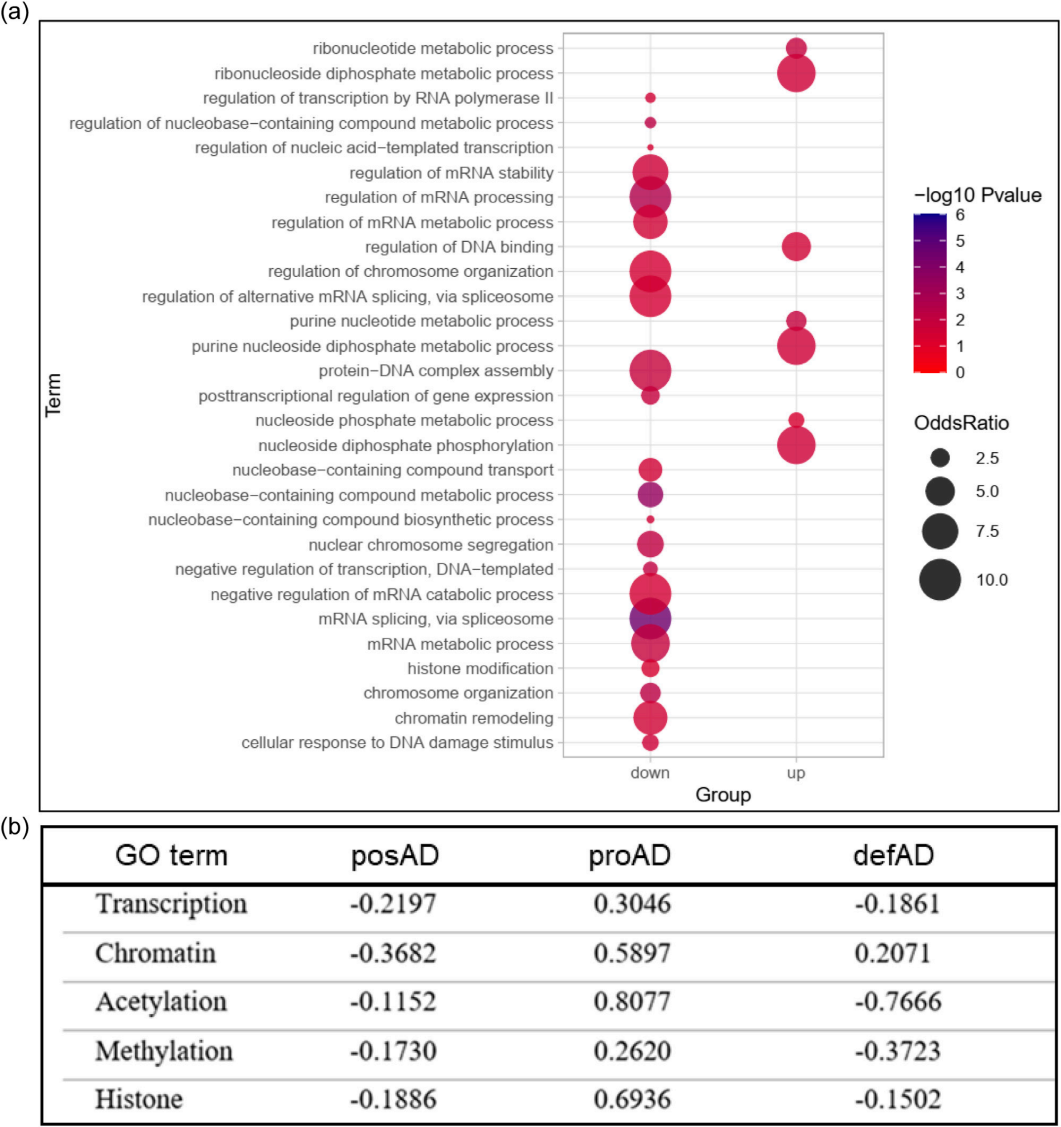
Chromatin remodeling pathways are altered by Tau. **(A)** GO analysis of proAD-mimic vs proAD common DEGs. **(B)** Correlation analysis of DEGs in chromatin remodeling pathways in posAD, proAD and defAD. Supplementary Table S4 for additional data.

GSEA analyses revealed that the main nuclear pathway altered by Tau is associated with chromatin remodeling and nucleosome assembly (Figure 1C). Due to the relevance of these mechanisms in gene expression regulation and genome stability, we investigated their impact in the meta-analysis with AD brains at different stages. We performed GO analyses of the common DEGs between the proAD-mimic and the human proAD dataset and we observed that most of the identified pathways are associated with nucleotide metabolism, transcription and chromatin remodeling (Figure 3A). To understand how these pathways are modulated in disease progression, we retrieved the genes with GO annotation terms related to chromatin remodeling, transcriptional and epigenetic regulation (Transcription, Chromatin, Acetylation, Methylation, Histone) (Supplementary Table S4), and we calculated their correlation between proAD-mimic and AD brains. Pathways related to transcription and chromatin modifications were uncorrelated or anticorrelated in the posAD and defAD stages, while we found a positive correlation in proAD (Figure 3B). This result suggests a key role for Tau in this temporal window of pathology and shows that the increase in total and soluble Tau can lead to the alteration of transcription networks (Kurbatskaya et al., 2016; Han et al., 2017). To independently detect networks of co-expressed genes that may be altered in disease progression, we employed a longitudinal weighted gene coexpression network analysis (WGCNA) on temporal lobe samples from both AD and healthy individuals. This unbiased analysis revealed two distinct modules related to nuclear functions and dysregulated in AD brains: the Pink and the Darkgrey modules (Supplementary Figure S4; Supplementary Table S6, S7). The GO analysis of these modules highlighted functions such as DNA binding and transcription regulation. These findings support the results obtained from meta-analyses, suggesting that pathways associated with chromatin and gene expression may indeed be affected during disease progression.

**FIGURE 4:**
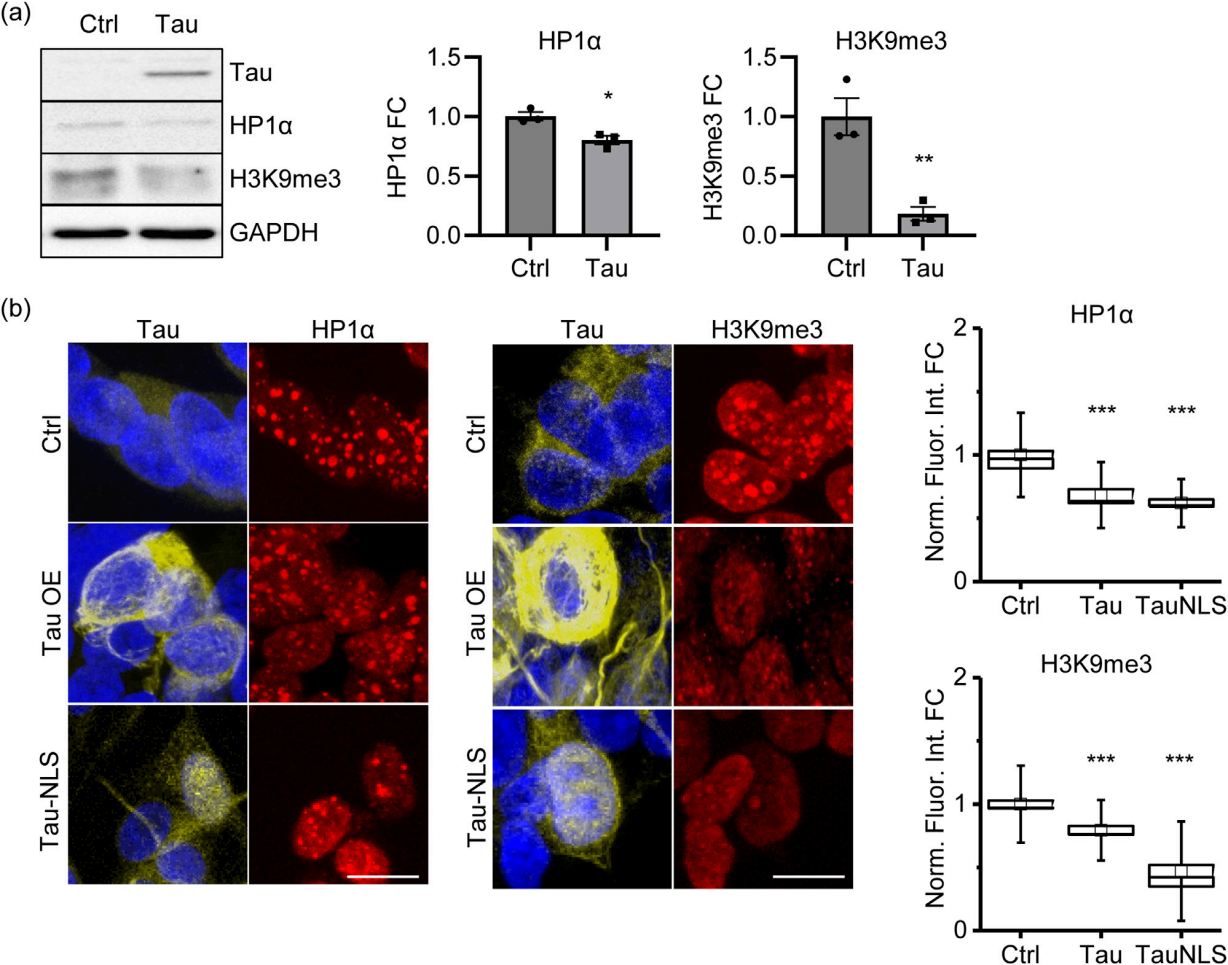
Tau induces a loss of heterochromatin markers. **(A)** WB of the heterochromatin markers HP1α and H3K9me3 in proAD-mimic (Tau) and control (Ctrl) cells. Relative quantification, bar plot of HP1α and H3K9me3 fold change in proAD-mimic (Tau) and control (Ctrl) cells. N = 3. **(B)** Immunofluorescence and relative quantification of HP1α and H3K9me3 in proAD-mimic and control cells. Red: HP1α/H3K9me3; yellow: Tau; blue: DAPI. (for HP1α analysis N = 60 in Ctrl cells; N = 30 in Tau overexpressing cells; N = 46 TauNLS) (for H3Kpme3 analysis N = 60 in Ctrl cells; N = 46 in Tau overexpressing cells; N = 68 TauNLS) Scale bar: 5 µm *p < 0.05; **p < 0.01.

Based on these data, we hypothesize that, in the early stages of the pathology, Tau might alter the expression of genes related to chromatin structure and organization, thus influencing genome stability and transcription. To elucidate chromatin remodeling deregulation, we detected the heterochromatin markers HP1α and H3K9me3, and we observed a significant reduction in the proAD-mimic compared to control cells by both immunoblotting (WB) (Figure 4A) and immunofluorescence (IF) (Figure 4B). These results indicate that increased Tau levels alter the amount of chromatin remodeling factors HP1α and H3K9me3, leading to a reduction in heterochromatin markers that mimics an early/intermediate AD condition. Of note, we observed that forcing the translocation of Tau (TauNLS) into the nuclear compartment still results in HP1α and H3K9me3 reduction and heterochromatin loss (Figure 4B).

**FIGURE 5:**
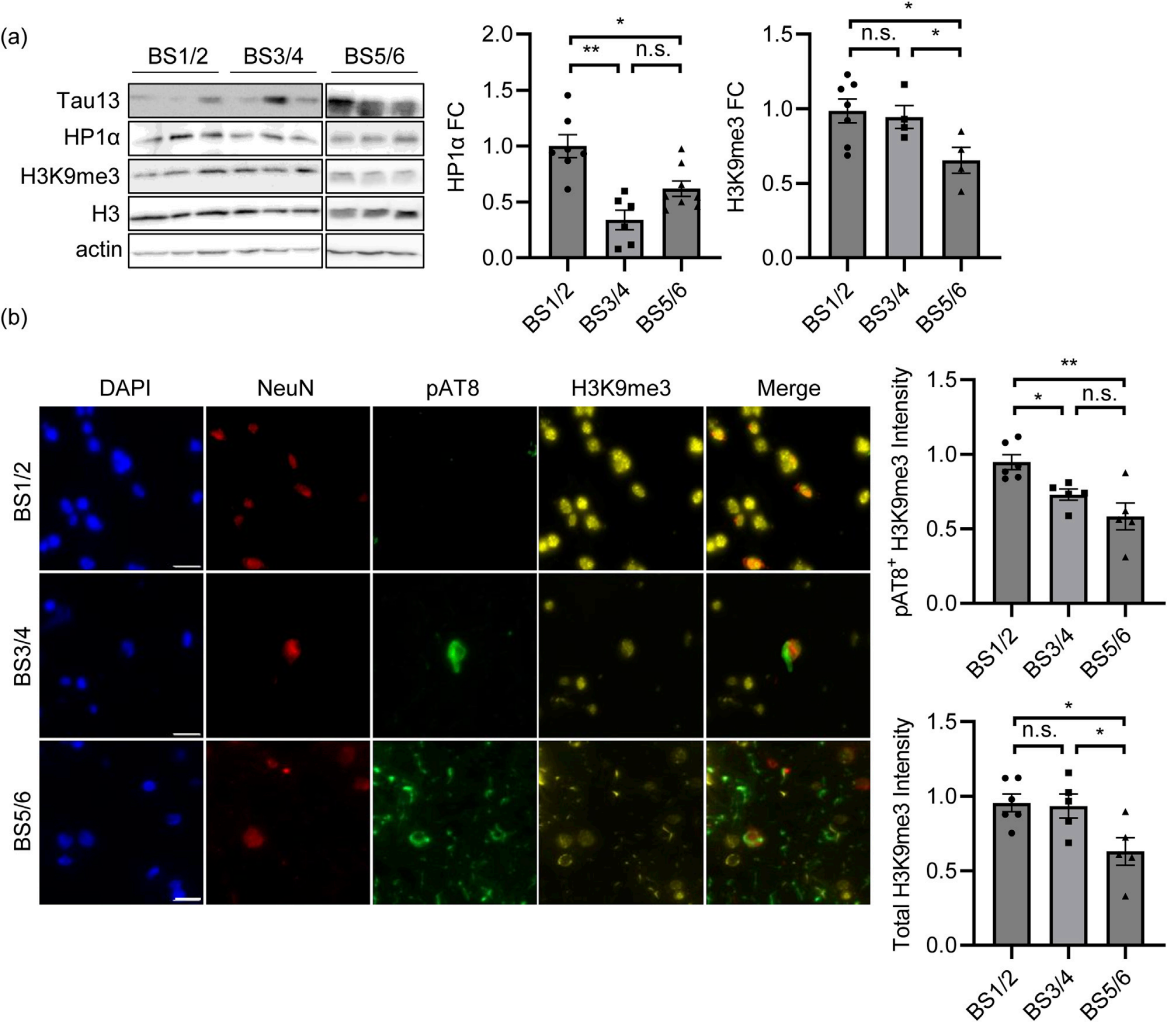
Heterochromatin changes are associated with Tau pathology in AD temporal lobes at intermediate stages. **(A)** WB of heterochromatin markers HP1α and H3K9me3 in human brain samples at BS1/2 (N = 7), BS3/4 (N = 6) and BS5/6 (N = 8) and relative quantification. Histone H3 and Actin are the housekeeping genes. HP1α and H3K9me3 fold change is reported in the graphs. H3K9me3 has been normalized on total H3 histone. **(B)** Immunofluorescence and relative quantification of H3K9me3 in human brain samples at BS1/2 (N = 6), BS3/4 (N = 5) and BS5/6 (N = 5). Red: NeuN; yellow: H3K9me3; green: pAT8; blue: DAPI. Scale bar: 20 µm *p < 0.05; **p < 0.01.

To confirm these results, we analyzed the temporal lobes of control (BS1/2) and AD patients at early/intermediate pathological stages. By WB analysis, we observed a significant reduction in HP1α but not H3K9me3 levels (Figure 5A) at Braak stages 3 and 4 (BS3/4) compared to those in controls (BS1/2). In IF experiments, we labelled AD temporal lobes with the Tau^AT8^ phospho-epitope as a marker of Tau pathology and NeuN as a marker of neuronal cells. At BS3 and BS4, we observed a sparse AT8 signal in a small percentage of NeuN^+^ cells. We analyzed the heterochromatin marker H3K9me3, and we observed that the total fluorescence was not altered as much as in the WB experiment. However, upon measuring the H3K9me3 signal in Tau^AT8^-positive neurons, we observed a significantly weaker signal than that in healthy brains (Figure 5B). Of note, at late AD stages (BS5/6), the AT8 signal was detected in all tissues as expected, and the total H3K9me3 fluorescence was significantly reduced, further supporting our evidence regarding the Tau-dependent alteration of heterochromatin (Figures 5A,B). This finding further supports the interplay between pathological Tau and heterochromatin changes in stages that anticipate the defAD condition.

### 3.4 Tau-dependent transcriptomic profile is associated with chromatin architecture markers in AD

To assess whether transcriptomic changes induced by Tau overexpression in the proAD-mimic model are associated to alterations in the chromatin state also observed in AD brains, we compared our DEGs to genes with changes in histone acetylation in AD patients. We gathered ChIP-seq data on the acetylation marks H3K27ac, H3K9ac and H3K122ac, associated to active enhancers and transcription, from four independent datasets of post-mortem AD brains (Marzi et al., 2018; Klein et al., 2019; Nativio et al., 2020; Patel et al., 2023). The datasets span different brain regions (entorhinal cortex, prefrontal cortex, lateral temporal lobe) from AD patients and matched controls, for a total of 764 donors, 457 of whom had AD.

**FIGURE 6:**
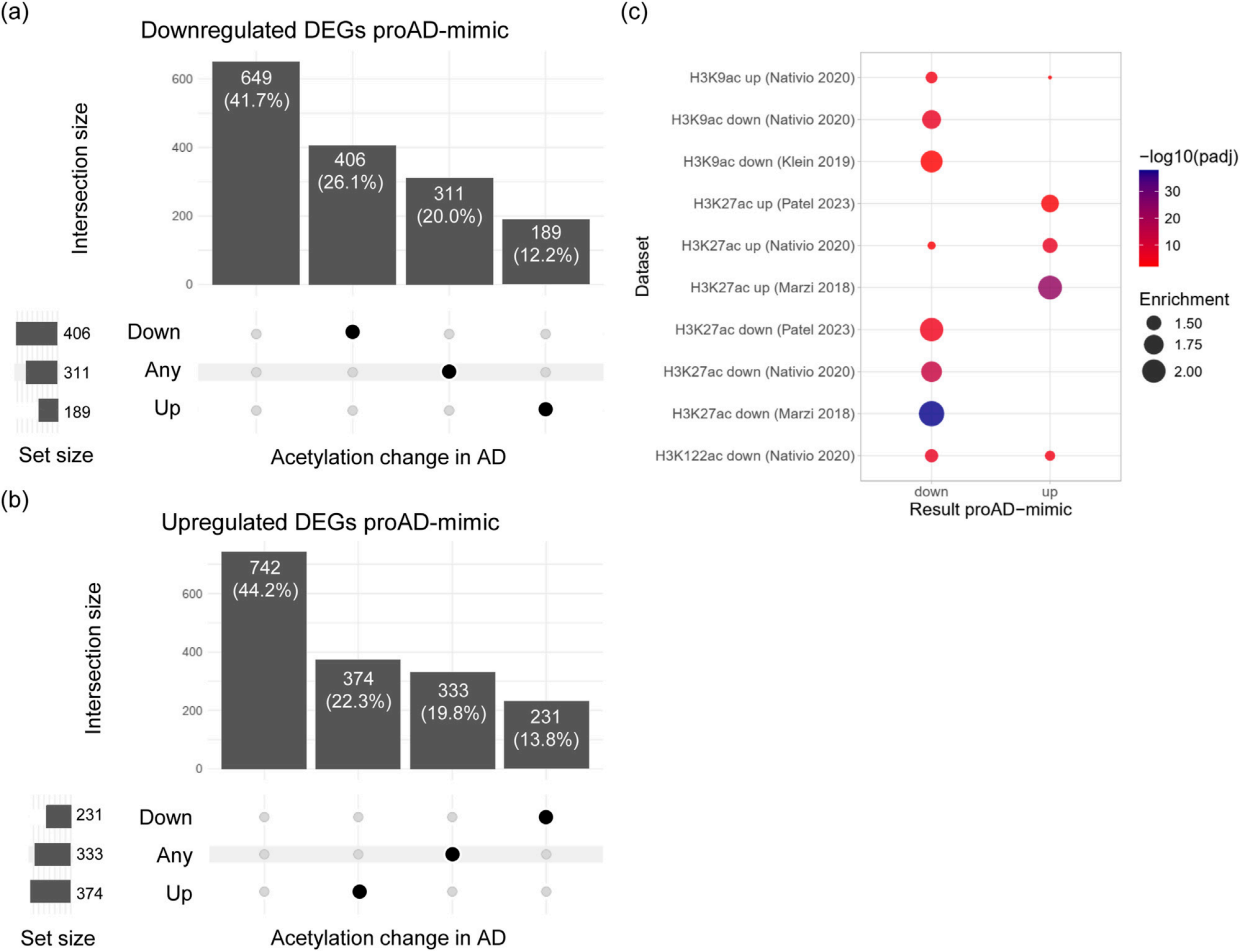
DEGs in the proAD-mimic transcriptome show changes in chromatin state in AD brains. **(A)** Upset plot displaying the overlap of downregulated DEGs in proAD-mimic condition with genes associated to changes in acetylation in AD. The overlap is reported as the number of genes in the exclusive intersection and their percentage over the total downregulated DEGs. **(B)** Upset plot showing the overlap of upregulated DEGs in proAD-mimic condition with genes associated to changes in acetylation in AD. The number of genes and their percentage over the upregulated DEGs are reported for each intersection. **(C)** Overrepresentation analysis of increased and decreased acetylation markers from different AD brain datasets in up- and downregulated DEGs from the proAD-mimic transcriptome. The point size is proportional to the enrichment, and the color to the BH-adjusted *p* value of the hypergeometric test.

We classified genes into three categories according to the change in acetylation across all datasets: ‘down’ if acetylation decreased in all datasets reporting a change (N = 637), ‘up’ if it increased (N = 563), and ‘any’ if the direction of change was inconsistent across datasets (N = 644). Among the downregulated DEGs, 58.3% were associated with acetylation changes in at least one dataset, and the majority (26.1%) showed a decrease in acetylation, consistent with reduced transcription (Figure 6A). Similarly, 55.8% of the upregulated DEGs had a change in acetylation in AD, mainly an increase (22.3%) (Figure 6B). Therefore, differential genes show changes in acetylation consistent with the change in transcription, suggesting that they could be correlated. A small proportion of genes (12.1% for down- and 13.8% for upregulated DEGs) displayed an opposite change in acetylation, potentially due to differences between the cell line model and the brain tissue, and to the early AD stage of the model *versus* the late AD stage of the patient-derived sample.

To test if the proportion of genes with acetylation changes among the proAD-mimic DEGs is higher than expected by chance, we performed an overrepresentation analysis of the differentially acetylated genes from AD brain datasets in the proAD-mimic up- and downregulated DEGs (Figure 6C). Genes with reduced acetylation on H3K27, H3K122 and H3K9 were overrepresented among the downregulated proAD-mimic DEGs, while genes with increased acetylation were generally enriched in the upregulated DEGs, confirming the concordance between chromatin state and transcription. Increased H3K9ac from Nativio et al., 2020 was the only exception, probably due to differences between the cell line model and the brain tissue, to the early AD stage of the model *versus* the late AD stage of the human sample, and to the technical limitations. Overall, transcriptional changes in the proAD-mimic model are associated with alterations in the chromatin state in AD patients and display similar trends, suggesting that our model reflects epigenetic and transcriptional changes in the AD brain.

## 4 Discussion

In addition to the current literature’s emphasis on Tau and chromatin remodeling in late stages, our study introduces a novel finding: Tau is actively involved in transcriptomic alterations in the early stages of Alzheimer’s disease pathology. This discovery sheds light on the disease progression and provides valuable insights for potential therapeutic interventions. Indeed, we found that Tau-dependent loss of heterochromatin markers occurs before irreversible neuronal damage and the onset of definitive AD. Building upon our prior findings regarding the role of pathological Tau in glutamatergic gene expression alterations, we investigated the connection between Tau and global transcriptional changes during AD progression (Siano et al., 2019b; Siano et al., 2020b). The increased availability of Tau led to changes in synaptic genes and a broader transcriptomic rearrangement (Siano et al., 2021), consistent with findings in other models, supporting that Tau-dependent gene modulation is an intrinsic function of Tau (Elrod et al., 2019; Montalbano et al., 2021). The GO and GSEA analyses of the transcriptome in the cellular model identified terms related to synaptic functions, as expected (Siano et al., 2019b; Siano et al., 2020b). Additionally, we found terms that are relevant for neuronal physiology and pathology, such as protein metabolism and immune system, and less canonical pathways, such as RNA metabolism or extracellular matrix remodeling (Violet et al., 2014; Hernández-Ortega et al., 2016; Chauderlier et al., 2018; Ma et al., 2020). To functionally validate our analysis, we measured the phosphorylation of eIF2α and we observed a significant increase of this well-known marker of response to ER stress and translational mechanisms (Clemens, 2001; Radford et al., 2015; Gerakis and Hetz, 2018; Shacham et al., 2021). Remarkably the GSEA analysis highlights nuclear pathways specifically associated with chromatin remodeling, suggesting that Tau altered levels could affect this function.

We conducted a meta-analysis to explore Tau-dependent pathways in AD progression. We compared the transcriptome of the *in vitro* model with datasets from brains at three AD stages. Notably, we observed an overlap of differentially expressed genes (DEGs) with the proAD stage. This finding supports the hypothesis that gene expression changes induced by Tau reflect a pathological condition preceding late AD phases. Consequently, we designated our cellular model as ‘proAD-mimic.’

A gene-by-gene FC comparison indicated a positive correlation between the proAD-mimic and proAD brains, indicating that the transcriptomic modifications observed in patients might be also mediated by Tau pathology, as previously suggested (Siano et al., 2021). Indeed, in proAD conditions, the Tau protein is destabilized, oligomerized, and highly soluble (Alonso et al., 1997; Guo et al., 2017; Lowe et al., 2018). Furthermore, the total and soluble amounts of Tau are significantly elevated in intermediate stages, especially in frontal and temporal brain regions, suggesting that the increase in Tau level can impair neuronal homeostasis and drive the pathological changes observed in late phases (Kurbatskaya et al., 2016; Han et al., 2017). In this context, several functions are compromised, such as synaptic transmission and DNA repair, and our findings suggest that these pathways could be transcriptionally altered by Tau (Colnaghi et al., 2020; Siano et al., 2021). Notably, the meta-analysis identified the proAD mimic as a reliable model to resemble a developing intermediate stage (proAD) *in vitro*.

The specificity of the proAD-mimic was further strengthened by the comparison with posAD and defAD brains. The posAD and defAD transcriptomes were negatively correlated and not correlated with our dataset, respectively. The negative correlation with posAD might suggest early transcriptomic compensation to prevent Tau-dependent alterations and to maintain neuronal homeostasis. In contrast, the loss of correlation with the terminal stages could be the consequence of excessive transcriptomic and functional damage in proAD leading to global gene dysregulation. This evidence closely relates to our previous study on the human prefrontal cortex. We observed significant deregulation of synaptic genes mainly at Braak stages 3 and 4 (early or intermediate AD stage), and we hypothesized a causal link with destabilized and oligomerized Tau protein (Siano et al., 2020b). Our meta-analysis further confirmed that in early and intermediate pathological stages, there is a Tau-dependent transcriptomic alteration impinging on the synaptic pathways.

We previously demonstrated that the imbalance of Tau in the nucleus causes alterations in glutamatergic genes. Here, we found that Tau alters other neuronal pathways associated with stages preceding severe AD pathology that are characterized by higher Tau levels in turn (Kurbatskaya et al., 2016; Han et al., 2017). Remarkably, by GSEA analysis on the proAD-mimic, we observed nuclear pathways exclusively associated with chromatin remodeling. These data are further supported by the employment of WGCNA analysis on AD brain samples, an unbiased technique that shows, among several pathways, loss of co-regulation of terms associated with DNA binding and transcription regulation during AD progression. Due to the increasing interest in alternative functions of Tau and the still scarce literature about its nuclear role, we examined the chromatin and transcriptional pathways emerging from the meta-analysis. The GO analysis of common genes in proAD-mimic and AD brains dataset identified several terms related to transcription and chromatin remodeling. The correlation analysis of epigenetic terms showed a positive correlation with proAD, as expected, whereas a negative correlation and no regulation were observed in posAD and defAD stages, respectively. The Tau-dependent alteration of chromatin remodeling pathways associated with proAD supports an early modification of chromatin structures (Eissenberg and Elgin, 2014; Frost et al., 2014; El Hajjar et al., 2019; Napoletano et al., 2021). Of note, the negative correlation with posAD suggests a Tau-dependent mechanism leading to a homeostatic compensative balance. The reductions of heterochromatin markers, HP1α and H3K9me3, in the proAD-mimic indicated Tau as a major factor leading to the loss of heterochromatin. We confirmed this evidence in human temporal tissues. At early/intermediate stages, H3K9me3 signal is specifically reduced in neurons that exhibit hyperphosphorylation of Tau at AT8 epitope, commonly associated with Tau pathology (Neddens et al., 2018), according with our previous observations. However, HP1α showed a stronger and wider reduction than H3K9me3. It is conceivable that alteration of HP1α, a general marker of heterochromatin, could be more responsive to minor Tau perturbations or that other mechanisms might have contributed. These data imply that Tau can affect chromatin pathways and cause heterochromatin loss in intermediate AD stages, thus influencing transcriptional mechanisms. In this context, a few specific evidences show the involvement of nuclear Tau in mechanisms associated with gene expression, such as the interaction of Tau with: i) TRIM28, to preserve the genomic stability (Rousseaux et al., 2016); ii) KDM6B, to regulate the expression of glutamatergic genes (Wang et al., 2022); iii) histones, to confer genotoxic resistance (Rico et al., 2021). Furthermore, we previously reported a competitive relationship between Tau and HDAC1 in the nuclear compartment leading to the pathological alteration of synaptic genes expression (Siano et al., 2023). Here, we found that nuclear Tau participates in the modulation of chromatin architecture, suggesting that it might be involved in the early pathological signature in AD.

The early alterations of HP1α and H3K9me3 could affect chromatin structure, thus enhancing transcriptional deregulation with a positive feedback mechanism. It is noteworthy that our *in vitro* system is sensitive to the modulation of Tau leading to transcriptome alterations and chromatin reorganization in earlier steps of the disease, suggesting that Tau dysfunction might be hierarchically upstream via direct interaction with chromatin, chromatin remodelers, or transcription factors or indirectly via modification of cofactor function. Further analysis, such as ChIP-sequencing and Hi-C could identify genomic loci and mechanisms involved in this dysregulation. Here, we reported the meta-analysis comparing the proAD-mimic transcriptome with several ChIP-seq databases of chromatin markers in AD brains. Consistently, the up- and downregulated genes fall in open or closed chromatin regions, respectively. The changes in chromatin structure and the concomitant transcriptomic alteration might feed into a self-perpetuating mechanism, causing a progressive disruption of transcriptome homeostasis and neuronal functions, which drives neurodegeneration. Nevertheless, based on our findings related to protein homeostasis disruption, it remains possible that some of the transcriptional changes are influenced by translational pathways. In addition, it has been previously reported that different Tau isoforms are able to translocate in the nucleus with different efficiencies, providing further insights about the effect of nuclear Tau on transcriptional and chromatin remodeling alterations (Liu and Götz, 2013). Our data suggest that neuronal damage caused by pathological Tau occurs and must be prevented early by targeting cellular mechanisms associated with transcription regulation. Beside the value of developing an *in vitro* model to study Tau-dependent genome modulation, it would be interesting to investigate the impact and extent of Tau-driven pathology on other brain cell types such as astrocytes and microglia.

## Data Availability

The datasets presented in this study can be found in online repositories. The names of the repository/repositories and accession number(s) can be found at: https://www.ncbi.nlm.nih.gov/, GSE239956, https://www.ncbi.nlm.nih.gov/geo/query/acc.cgi?acc=GSE239956.
