# Supplementary material for: Tau mediates the reshaping of the transcriptional landscape toward intermediate Alzheimer’s disease stages
Source: Front Cell Dev Biol. 2025 Jan 3;12:1459573. doi: 10.3389/fcell.2024.1459573 (PMC11739074; doi:10.3389/fcell.2024.1459573)
Supplement: Supplementary file 3 [file DataSheet4.pdf]

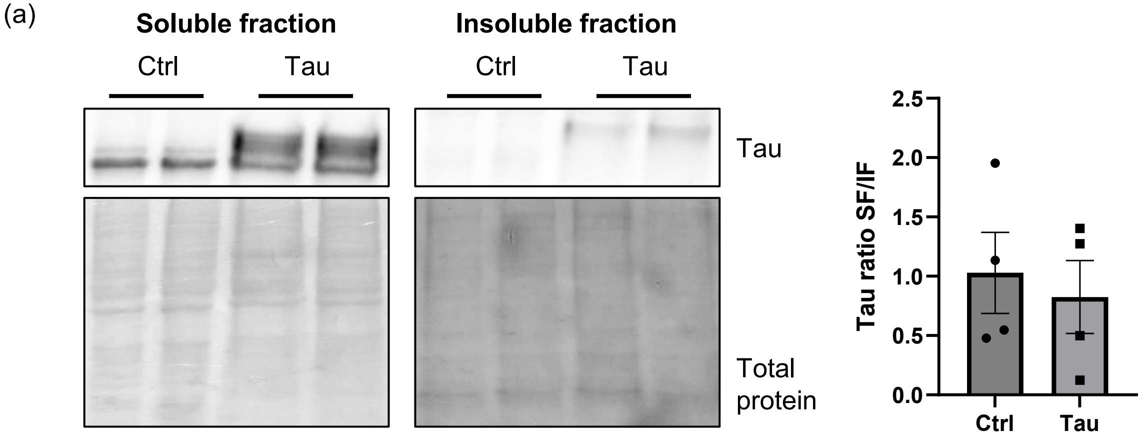

Suppl. Fig 3. Overexpressed Tau is as soluble as the endogenous one. Western blot analyses of soluble and insoluble fractions obtained by subcellular fractionation performed as in Siano et al 2019, and relative quantification. N=4.
