# Supplementary material for: Tau mediates the reshaping of the transcriptional landscape toward intermediate Alzheimer’s disease stages
Source: Front Cell Dev Biol. 2025 Jan 3;12:1459573. doi: 10.3389/fcell.2024.1459573 (PMC11739074; doi:10.3389/fcell.2024.1459573)
Supplement: Supplementary file 5 [file DataSheet3.pdf]

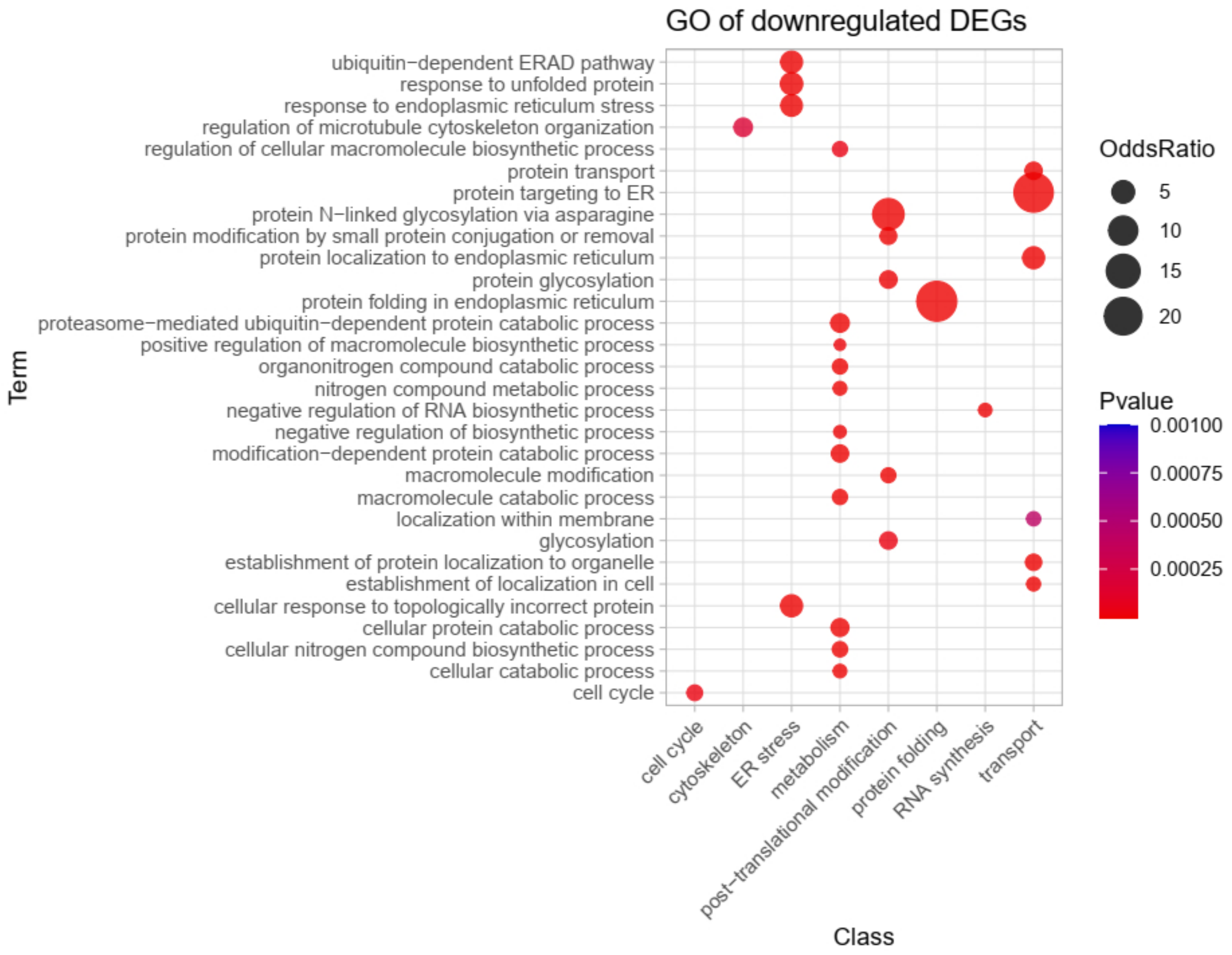

Suppl. Fig 2. GO overrepresentation analysis of downregulated pathways. GO for biological process was done on unregulated and down regulated DEGs separately. The most enriched terms from the GO for biological process, on the vertical axis, were classified into broader classes, which are plotted on the horizontal axis. The radius of the dot is proportional to the enrichment, and the colour to the P value. Supplementary Table 3 for additional data.
