# Supplementary material for: Tau mediates the reshaping of the transcriptional landscape toward intermediate Alzheimer’s disease stages
Source: Front Cell Dev Biol. 2025 Jan 3;12:1459573. doi: 10.3389/fcell.2024.1459573 (PMC11739074; doi:10.3389/fcell.2024.1459573)
Supplement: Supplementary file 7 [file DataSheet1.pdf]

Supplementary Materials and methods

Preparation of TritonX 100-Insoluble Fractions

Cells were lysed with lysis buffer (1% Triton-X 100 in PBS with protease and phosphatase inhibitors) and the extract was centrifuged at 16000 × *g* for 15 min. The supernatant was designated as Triton X-100 soluble fraction. The pellet was dissolved in the canonical lysis buffer (1% SDS; 1% Triton-X in PBS with protease and phosphatase), sonicated and boiled. This fraction was designated to be Triton X-100 insoluble fraction.

Real-time PCR

Total RNA was extracted by Nucleospin (Macherey-Nagel) and retro-transcribed by Reverse Transcriptase Core kit (Eurogentec) according to the manufacturer's instructions. Real-time PCR was performed using the iTaqTM Universal SYBR® Green Supermix (BioRad) and performed for 40 cycles of amplification with denaturation at 95 °C for 15 s, annealing at 60 °C for 25 s and extension at 72 °C for 20 s. The primers employed were as follows:
actin: fwd 5'-TCCATCCTGGCCTCACTGTCCAC-3', rev 5'-GAGGGGCCGGACTCATCGTACT-3';
kat2a: fwd 5’-GAGGTCATGCTGACCATCACTG-3’, rev 5’ -CAGTGAGTTGCCGATGACATGG-3’;
txnip:_fwd 5’-CAGCAGTGCAAACAGACTTCGG-3’, rev 5’-CTGAGGAAGCTCAAAGCCGAAC-3’;
pparg: fwd 5’-AGCCTGCGAAAGCCTTTTGGTG-3’, rev 5’-GGCTTCACATTCAGCAAACCTGG-3’;
prmt6: fwd 5’-GGAGCTGGAGGCCGGAGTGGG-3’, rev 5’-GCGTGATCTCTCCTGAAACGT-3’;
tgm2: fwd 5’-TGTGGCACCAAGTACCTGCTCA-3’, rev 5’-GCACCTTGATGAGGTTGGACTC-3’.

WGCNA

For WGCNA, the temporal region data on the Affymetrix Human Genome U133B Array (GPL97) platform from the GSE84422 dataset was used. The dataset was divided into subsets according to the AD stage (posAD, proAD, defAD). WGCNA was performed with the R package WGCNA v 1.72.5 (Langfelder and Horvath, 2008) and consensus networks were built as described in Miller, J., “Meta-analyses of data from two (or more) microarray datasets”, https://labs.dgsom.ucla.edu/file/25953/Tutorial_document.pdf., using β = 9 to fit a scale-free distribution. Modules were built on the control set using the signed adjacency matrix as the distance measure, setting deepSplit = 3 and cluster size 30-3*deepSplit. Module conservation was assessed by Z-summary: > 10 for highly conserved modules, < 2 for not conserved modules. PosAD was not displayed because all the healthy control modules were conserved. To choose the appropriate number of clusters, the modules were inspected visually in Cytoscape and with assignment indicators with the R packages ppclust v1.1.0.1 (Cebeci, 2019) , factoextra v1.0.7 (Kassambara and Mundt, 2020), cluster v2.1.4 (Maechler M, Rousseeuw P, Struyf A, Hubert M, 2023) and fclust v2.1.1.1 (Ferraro et al., 2019), and then the modified partition coefficient and the partition entropy were maximized. To associate each cluster with a molecular function, gProfiler software (Reimand et al., 2007) was used, including all known genes in the GO database. Top pathways with Benjamini–Hochberg FDR values < 0.05 defined cluster identity.

References

Cebeci, Z. (2019). Comparison of Internal Validity Indices for Fuzzy Clustering. *J. Agric. Informatics* 10. doi: 10.17700/jai.2019.10.2.537

Ferraro, M. B., Giordani, P., and Serafini, A. (2019). Fclust: An R package for fuzzy clustering. *R J.* 11, 1–18. doi: 10.32614/rj-2019-017

Kassambara, A., and Mundt, F. (2020). factoextra: Extract and Visualize the Results of Multivariate Data Analyses. Available at: https://cran.r-project.org/package=factoextra

Langfelder, P., and Horvath, S. (2008). WGCNA: an R package for weighted correlation network analysis. *BMC Bioinformatics* 9, 559. doi: 10.1186/1471-2105-9-559

Maechler M, Rousseeuw P, Struyf A, Hubert M, H. K. (2023). cluster: Cluster Analysis Basics and Extensions. Available at: https://cran.r-project.org/package=cluster

Reimand, J., Kull, M., Peterson, H., Hansen, J., and Vilo, J. (2007). g:Profiler—a web-based toolset for functional profiling of gene lists from large-scale experiments. *Nucleic Acids Res.* 35, W193–W200. doi: 10.1093/nar/gkm226
