# Supplementary material for: Tau mediates the reshaping of the transcriptional landscape toward intermediate Alzheimer’s disease stages
Source: Front Cell Dev Biol. 2025 Jan 3;12:1459573. doi: 10.3389/fcell.2024.1459573 (PMC11739074; doi:10.3389/fcell.2024.1459573)
Supplement: Supplementary file 8 [file DataSheet5.pdf]

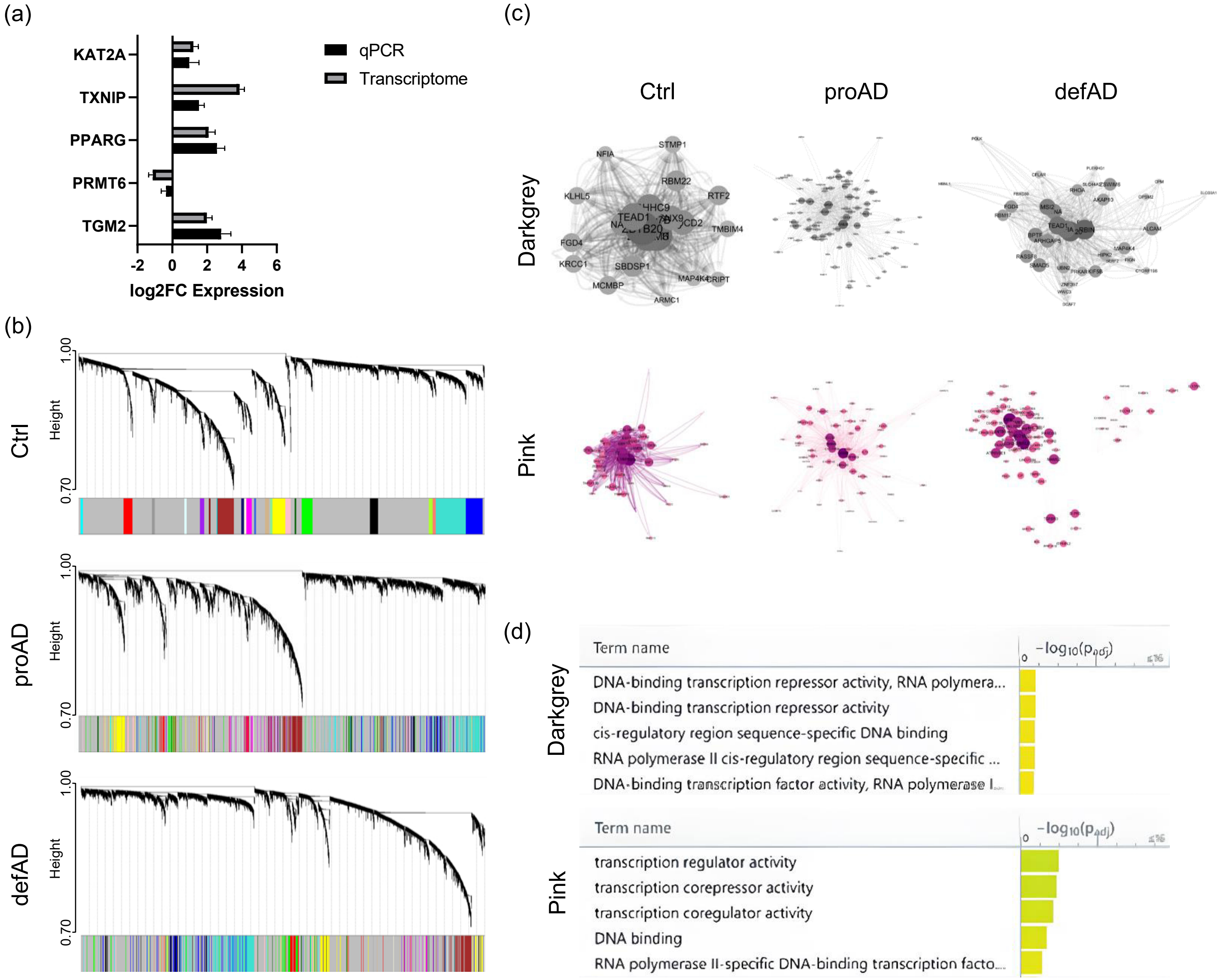

Suppl. Fig 4. Network analysis of the AD temporal cortex shows a progressive alteration of transcriptomic pathways. (A) Bar plot of selected genes analysed by qPCR and RNA-seq. (B) WGCNA coregulation analysis of control (Ctrl), proAD and defAD human temporal lobe. (C) Network representation of genes of the Darkgrey and Pink modules in Ctrl, proAD and defAD brains. Supplementary Table 5 for additional data. (D) Transcription-related terms in the GO analysis of the Darkgrey and Pink modules. Top 5 transcription-related GO pathways have been reported. Supplementary Table 6-7 for additional data.
